# Alcohol consumption, substance use, and depression in relation to HIV Pre-Exposure Prophylaxis (PrEP) nonadherence among gay, bisexual, and other men-who-have-sex-with-men

**DOI:** 10.1186/s12889-020-09883-z

**Published:** 2020-11-25

**Authors:** Paul A. Shuper, Narges Joharchi, Isaac I. Bogoch, Mona Loutfy, Frederic Crouzat, Philippe El-Helou, David C. Knox, Kevin Woodward, Jürgen Rehm

**Affiliations:** 1 Institute for Mental Health Policy Research & Campbell Family Mental Health Research Institute, Centre for Addiction and Mental Health (CAMH), 33 Russell St., Toronto, ON M5S 2S1 Canada; 2 Dalla Lana School of Public Health, University of Toronto, 155 College St., Toronto, ON M5T 3M7 Canada; 3 Divisions of General Internal Medicine and Infectious Diseases, University Health Network, 200 Elizabeth St., Toronto, ON M5G 2C4 Canada; 4 Department of Medicine, University of Toronto, Medical Sciences Building, 1 King’s College Circle, Toronto, ON M5S 1A8 Canada; 5 Women’s College Hospital, 76 Grenville St., Toronto, ON M5S 1B2 Canada; 6 Maple Leaf Medical Clinic, 14 College St., Toronto, ON M5G 1K2 Canada; 7 Department of Medicine, Division of Infectious Diseases, McMaster University, 1200 Main St. W., Hamilton, ON L8N 3Z5 Canada; 8 Department of Family and Community Medicine, University of Toronto, 500 University Ave., Toronto, ON M5G 1V7 Canada; 9 Department of Psychiatry, University of Toronto, 250 College St., Toronto, ON M5T 1R8 Canada; 10 PAHO/WHO Collaborating Centre for Addiction and Mental Health, 33 Russell St., Toronto, ON M5S 2S1 Canada; 11 Epidemiological Research Unit, Technische Universität Dresden, Chemnitzer Str. 46, 01187 Dresden, Germany; 12 Klinische Psychologie & Psychotherapie, Chemnitzer Str. 46, 01187 Dresden, Germany; 13 Department of International Health Projects, Institute for Leadership and Health Management, I.M. Sechenov First Moscow State Medical University, 8-2 Trubetskaya str., Moscow, Russian Federation 119991; 14 Graduate Department of Community Health and Institute of Medical Science, University of Toronto, Medical Sciences Building, 1 King’s College Circle, Toronto, ON M5S 1A8 Canada

**Keywords:** HIV pre-exposure prophylaxis (PrEP), MSM, Adherence, Alcohol, Substance use, Depression

## Abstract

**Background:**

Although HIV pre-exposure prophylaxis (PrEP) substantially diminishes the likelihood of HIV acquisition, poor adherence can decrease the HIV-protective benefits of PrEP. The present investigation sought to identify the extent to which alcohol consumption, substance use, and depression were linked to PrEP nonadherence among gay, bisexual, and other men-who-have-sex-with-men (gbMSM).

**Methods:**

gbMSM (age ≥ 18, prescribed PrEP for ≥3 months) were recruited from two clinics in Toronto, Canada for an e-survey assessing demographics; PrEP nonadherence (4-day PrEP-focused ACTG assessment); hazardous and harmful alcohol use (AUDIT scores of 8–15 and 16+, respectively); moderate/high risk substance use (NIDA M-ASSIST scores > 4); depression (CESD-10 scores ≥10); and other PrEP-relevant factors. The primary outcome, PrEP nonadherence, entailed missing one or more PrEP doses over the past 4 days. A linear-by-linear test of association assessed whether increasing severity of alcohol use (i.e., based on AUDIT categories) was linked to a greater occurrence of PrEP nonadherence. Univariate logistic regression was employed to determine factors associated with PrEP nonadherence, and factors demonstrating univariate associations at the *p* < .10 significance level were included in a multivariate logistic regression model. Additive and interactive effects involving key significant factors were assessed through logistic regression to evaluate potential syndemic-focused associations.

**Results:**

A total of 141 gbMSM (Mean age = 37.9, white = 63.1%) completed the e-survey. Hazardous/harmful drinking (31.9%), moderate/high risk substance use (43.3%), and depression (23.7%) were common; and one in five participants (19.9%) reported PrEP nonadherence. Increasing alcohol use level was significantly associated with a greater likelihood of nonadherence (i.e., 15.6, 25.0, and 44.4% of low-risk, hazardous, and harmful drinkers reported nonadherence, respectively (χ^2^(1) = 4.79, *p* = .029)). Multivariate logistic regression demonstrated that harmful alcohol use (AOR = 6.72, 95%CI = 1.49–30.33, *p* = .013) and moderate/high risk cocaine use (AOR = 3.11, 95%CI = 1.01–9.59, *p* = .049) independently predicted nonadherence. Furthermore, an additive association emerged, wherein the likelihood of PrEP nonadherence was highest among those who were hazardous/harmful drinkers and moderate/high risk cocaine users (OR = 2.25, 95%CI = 1.19–4.25, *p* = .013). Depression was not associated with nonadherence.

**Conclusions:**

Findings highlight the need to integrate alcohol- and substance-focused initiatives into PrEP care for gbMSM. Such initiatives, in turn, may help improve PrEP adherence and reduce the potential for HIV acquisition among this group.

## Background

Human immunodeficiency virus (HIV) persists as a public health issue, and within this context, gay, bisexual, and other men who have sex with men (gbMSM) continue to be disproportionately impacted by the virus compared to the population in general [1–3]. Of particular concern is that the decline in overall HIV incidence in countries such as the United States and Canada has not been reflected in corresponding populations of gbMSM, among whom HIV incidence has remained steady, and in some cases, has even increased [1–3].

In recent years, biomedical strategies have moved to the forefront of HIV prevention, with HIV pre-exposure prophylaxis (PrEP) - entailing a daily dose of tenofovir disoproxil fumarate/emtricitabine (TDF/FTC) or tenofovir alafenamide/emtricitabine (TAF/FTC) - offering non-infected individuals an effective method of protection from the virus. Empirical support for PrEP has been strong, with evidence from randomized controlled trials (RCTs) and real-world demonstration studies showing a reduction in the rate of HIV infection by 44–99% [4–10]. While this suggests that implementing PrEP on a broad scale could meaningfully reduce incident HIV among populations of gbMSM, PrEP’s ability to prevent HIV has been shown to be directly contingent upon adherence. In fact, across a number of PrEP-focused RCTs that reported ranges of adherence from 29 to 98%, the failure to adhere to PrEP was recognized as the most substantial hindrance to its effectiveness [11]. For example, in a seminal PrEP trial [5], PrEP-prescribed participants with non-detectable medication levels in their system were 13 times more likely to become infected with HIV than those with detectable levels; indicating that poor adherence diminished PrEP’s HIV preventive effect (see also [7, 8, 12–14]). While some evidence suggests that on-demand PrEP (i.e., taking PrEP only in conjunction with sexual events) or intermittent PrEP (i.e., less frequent, non-daily dosing schedules) may provide a fair degree of protection [7, 9, 12, 15], given the marked adherence-related outcomes demonstrated across multiple PrEP RCTs, and recognizing some of the potential complexities surrounding non-daily PrEP dosing [16–19], PrEP guidelines have tended to maintain their impetus for daily PrEP adherence [20, 21].

### Alcohol consumption and adherence to PrEP

Compared to the general population, alcohol-related issues have been shown to be disproportionately elevated among populations of gbMSM [22–25] as well as individuals who have been prescribed PrEP [26, 27]. Notably, such issues may be particularly detrimental to adherence. It has been theorized that consuming alcohol can limit the cognitive capacity required to remember to follow one’s regimen as prescribed [28, 29], and in accord with this supposition, alcohol has repeatedly been associated with poor medication adherence [30], with much of this evidence derived from investigations on adherence to antiretroviral therapy (ART) among gbMSM living with HIV [29, 31]. More recently, a small number of studies have investigated alcohol’s association specifically with adherence to PrEP; however, findings from this work have been mixed. Haberer et al. [32] found that individuals exhibiting heavy drinking patterns as identified through the validated Rapid Alcohol Problems Screen [33] were approximately three times more likely to be suboptimally adherent to PrEP (i.e., < 80% adherent based on unannounced pill counts) than those who did not consume alcohol at heavy levels. Similarly, Mugo et al. [34] reported that having sex “while drunk” was shown to be marginally associated (*p* = .06) with lower adherence to one’s PrEP daily dosing regimen (assessed as a continuous measure via medication event monitoring system). These patterns have been further supported through qualitative research, in which alcohol consumption was perceived to hinder one’s ability to take PrEP as prescribed [35]. Diverging from these findings are those from Grove et al. [36], who found no link between heavy drinking during the past 30 days (i.e., ≥5 drinks in one sitting) and self-reported PrEP adherence among a sample of gbMSM (half of whom were club drug users); and from Hojilla et al. [37], who found that recent binge drinking (i.e. ≥5 drinks in a single day) was not significantly related to PrEP adherence as defined by dried blood spot tenofovir diphosphate concentrations. Of further divergence are the results from Velloza et al. [38], who demonstrated that compared to non-heavy drinking PrEP-users, those who reported heavy alcohol use (i.e., Alcohol Use Disorders Identification Test (AUDIT) score ≥ 8) had significantly higher hair, but not plasma, FTC/TFV concentrations. The authors, however, indicated that this elevated concentration may have been due to a pharmacokinetic effect of alcohol on FTC/TFV levels.

Potentially contributing to the complexity of the alcohol and PrEP nonadherence relationship is the high prevalence of concurrent substance use and depression among gbMSM. A recent investigation quantifying the extent of such comorbidities demonstrated that almost 40% of hazardous drinking gbMSM had been diagnosed with a substance use disorder (i.e., other than alcohol), and over a quarter had been diagnosed with a major depressive disorder [39] (see also [40]). Within the context of PrEP treatment, substance use and depression on their own have been linked to PrEP nonadherence, but similar to alcohol, disparate findings have been yielded. Specifically, nonadherence has been significantly associated with the use of some types or classes of substances (e.g., stimulants, club drugs) but not others (e.g., cannabis) [36, 37, 41–44]; and the presence of depressive symptomatology has been associated with both an increased and decreased likelihood of PrEP nonadherence among gbMSM [45].

Taken as a whole, it remains unclear whether inconsistent findings involving alcohol, substance use, and depression derive from the possible underlying confounding of these concurrent factors. Despite the prominent use of “syndemic” approaches in the ART literature that have examined the synergistic interplay of substance use- and mental health-related issues in relation to ART nonadherence [46, 47], such methodologies have typically been absent from the PrEP literature; thus limiting our knowledge about the extent to which alcohol, either alone or in combination with comorbid substance use and/or depression, is linked to missing one’s PrEP doses. The present investigation therefore employed an encompassing approach aimed at 1) evaluating alcohol’s possible independent association with PrEP nonadherence, and 2) exploring potential deleterious additive and interactive influences of alcohol in combination with concurrent substance use and depression on adherence behavior.

## Methods

### Participants, setting, and procedures

From August 2018 to February 2019, convenience sampling was employed to recruit participants from two clinics in downtown Toronto, Canada: one situated within a tertiary hospital and the other a stand-alone primary care community practice, that specialized in gbMSM- and HIV-focused medical services. Study eligibility criteria included: 1) ≥18 years of age; 2) receiving daily PrEP from one of the two clinics; 3) taking PrEP for at least 3 months; and 4) identifying as gay, bisexual, and/or as a man who has sex with other men. Our target sample size was 120 out of a population of approximately 650 potentially eligible individuals across the two clinics. Clinic staff referred men who were presenting for PrEP-related care to an on-site Research Assistant who described the study and obtained written consent. Participants completed a confidential, self-administered e-survey that assessed PrEP adherence and its potential correlates (see “Measures” below). The e-survey was programmed using Qualtrics [48] and was designed to be completed within 30 min. Participants received CAD $30 (~ USD $22.50) for taking part in the study. All procedures were approved by Research Ethics Boards at the Center for Addiction and Mental Health (Protocol# 101–2018) and the University Health Network (Protocol# 18–5014).

### Measures

#### Demographics

Items in the first section of the e-survey assessed age, race/ethnicity, level of education, and employment status. Participants were also asked about their current living situation and whether or not they presently had a steady partner.

#### PrEP adherence and duration on PrEP

The AIDS Clinical Trials Group (ACTG) 4-day adherence assessment [49] was adapted for the present investigation to measure adherence to one’s PrEP regimen. Participants were asked to indicate whether or not they had missed their PrEP dose during each of the past 4 days. Nonadherence was defined as missing one or more PrEP doses during that timeframe. Duration on PrEP, in months, was asked through a single self-report item.

#### Alcohol use

Consumption of alcohol during the past 12 months was assessed through the Alcohol Use Disorders Identification Test (AUDIT) [50]. The first item of the AUDIT, stating “How often do you have a drink containing alcohol?”, was used to classify participants as non-drinkers versus drinkers (i.e., “never” vs. all other responses, respectively). Scores based on the full 10-item measure were categorized in accordance with AUDIT-based criteria to classify participants’ consumption as “low risk” (scores 0–7), “hazardous” (scores 8–15), or “harmful” (scores ≥16) [50].

#### Substance use

The National Institute on Drug Abuse Modified Alcohol, Smoking and Substance Involvement Screening Test (NIDA M-ASSIST) [51] was employed to classify substance use. Substances that were queried included cannabis, cocaine, stimulants, methamphetamines, inhalants, sedatives, hallucinogens, street opioids, and prescription opioids. For each substance, NIDA M-ASSIST “substance involvement scores” were calculated to identify “Moderate-Risk” (scores 4–26) and “High-Risk” (scores 27+) use; reflecting consumption-related patterns and/or consequences that would warrant intervention. As only a very small number of participants met criteria for high-risk use (i.e., *n* = 3 out of *N* = 141), moderate- and high-risk classifications were aggregated for each substance and categorized accordingly.

#### Depression

The self-report Center for Epidemiologic Studies Depression 10-item scale (CESD-10) [52] was employed to assess depressive symptomatology, and participants with a CESD-10 score ≥ 10 were classified as depressed. A separate, single-item question designed for this study asked participants if they had received any treatment for depression (e.g., medication, counselling, psychotherapy) within the past 3 months.

#### Sexual behavior

A comprehensive assessment based on a validated, gbMSM-focused sexual behavior questionnaire [53] was adapted for this investigation. Participants were asked to indicate the number of HIV-negative, HIV status unknown, and HIV-positive partners (who were also delineated by perceived HIV viral load) with whom they had sex during the past 3 months. For each partner HIV serostatus category, participants were asked about the number of condom-protected and condomless receptive and insertive anal sexual acts that they had engaged in. Based on this assessment, dichotomous variables were created to classify participants in terms of those who engaged in condomless sex during the past 3 months versus those who did not; delineated by partner HIV serostatus.

### Statistical analysis

The primary outcome focused on PrEP nonadherence as assessed through the ACTG-based measure described above. Statistical analysis first entailed a linear-by-linear test of association to assess whether increasing severity of alcohol use (i.e., AUDIT-based classification) was linked to a greater occurrence of PrEP nonadherence. Univariate logistic regression was then employed to assess the extent to which alcohol use, risky substance use, depression, and other factors of potential relevance to PrEP treatment (e.g., demographics, duration on PrEP, sexual partnerships) were associated with PrEP nonadherence. Factors significant at *p* < .10 in univariate analyses were included in a multivariate logistic regression model to identify independent predictors of PrEP nonadherence.

Alcohol-, substance use-, and depression-related factors that were found to be significant in the multivariate logistic regression model were further examined for possible syndemic production through tests of additive and interactive effects on PrEP nonadherence. A coding scheme was developed whereby the presence of a specific factor (e.g., hazardous/harmful drinking) was coded as “1,” and the absence coded as “0.” Accordingly, tests for possible additive effects entailed a logistic regression equation in which each participant’s sum of factors was regressed on PrEP nonadherence. Tests for possible interactive, synergistic effects (e.g., determining whether the impact of alcohol plus moderate/high risk substance use on PrEP nonadherence exceeded the effects of the sum of the issues) followed procedures put forth by Tsai et al. [54, 55], and involved logistic regression modelling in which interaction terms between/among the factors were assessed in relation to PrEP nonadherence. Statistical analyses were conducted using SPSS version 24 [56].

## Results

### Sample characteristics

A total of 142 individuals consented to take part in the study. Data were excluded from one participant who did not complete the survey in its entirety, which yielded a sample of 141 for analysis. Sample characteristics can be found in Table 1. As shown in the table, mean age was 37.9, and most participants identified their race as white (63.1%) and sexual orientation as gay (92.9%). High levels of employment, education, and income were evident among the sample. Fewer than half of participants (39.0%) reported a current steady partnership, and the vast majority of the sample (93.6%) reported the engagement in condomless sex during the past 3 months. Mean duration on PrEP was 16.8 months, and one in five participants (19.9%) reported missing at least one PrEP dose during the past 4 days.

**Table 1:** Characteristics of study participants

	*N* = 141
Age: M (SD)	37.9 (10.6)
Race/ethnicity n (%)	
White	89 (63.1%)
Chinese	14 (9.9%)
South Asian	7 (5.0%)
Multi-race	7 (5.0%)
Black	5 (3.5%)
Arab	5 (3.5%)
Latin American	4 (2.8%)
Filipino	3 (2.1%)
West Asian	3 (2.1%)
Aboriginal	2 (1.4%)
Southeast Asian	1 (0.7%)
Japanese	1 (0.7%)
Gender: n (%)	
Male	139 (98.6%)
Non-binary	2 (1.4%)
Sexual Orientation: n (%)	
Gay	131 (92.9%)
Bisexual	8 (5.7%)
Queer	2 (1.4%)
Education = completed college/university: n (%)	114 (80.9%)
Employed full-time: n (%)	114 (80.9%)
Annual household income ≥ CAD $100,000: n (%)	63 (47.0%)
Currently have a steady partner: n (%)	55 (39.0%)
Engaged in condomless sex during the past 3 months with …: n (%)	
Any partner(s)	132 (93.6%)
Any HIV-negative partner(s)	109 (79.6%)
Any HIV status unknown partner(s)	88 (64.7%)
Any HIV-positive partner(s)	62 (44.9%)
Months on PrEP: M (SD)	16.8 (14.8)
PrEP nonadherence – past 4 days: n (%)	28 (19.9%)

Percentages are based on the number of participants who indicated a specific response divided by the number of participants who responded to the item

*CAD* Canadian Dollar, *PrEP* Pre-Exposure Prophylaxis

### Alcohol, substance use, and depression

As shown in Table 2, alcohol use was highly prevalent among the sample (drinkers = 92.9%). One quarter (25.5%) of all participants met AUDIT criteria for hazardous drinking, and an additional 6.4% of the sample met AUDIT criteria for harmful alcohol use. Substance use was also common, whereby 43.3% of participants were classified as moderate/high risk users of at least one substance. Approximately one quarter of the sample (23.7%) met CESD-10 criteria for depression, and approximately one in five participants (19.7%) reported receiving treatment for depression in the past 3 months.

**Table 2:** Alcohol, substance use, and depression among study participants

	*N* = 141
Alcohol consumption – any (AUDIT): n (%)	131 (92.9%)
Alcohol consumption – risk category (AUDIT): n (%)	
Low risk (score 0–7)	96 (68.1%)
Hazardous drinking (score 8–15)	36 (25.5%)
Harmful drinking (score ≥ 16)	9 (6.4%)
Substance use – moderate/high risk (NIDA M-ASSIST): n (%)	
Cannabis	44 (31.2%)
Cocaine	18 (12.8%)
Inhalants	10 (7.1%)
Sedatives	9 (6.4%)
Methamphetamines	6 (4.3%)
Hallucinogens	6 (4.3%)
Stimulants	2 (1.4%)
Prescription opioids	1 (0.7%)
Street opioids	0 (0.0%)
Any substance	61 (43.3%)
Depression-related indicators	
Currently experiencing depression (CESD-10 score ≥ 10): n (%)	33 (23.7%)
Received treatment for depression – past 3 months: n (%)	25 (19.7%)

Percentages are based on the number of participants who indicated a specific response divided by the number of participants who responded to the item

*AUDIT* Alcohol Use Disorders Identification Test, *NIDA M-ASSIST* National Institute on Drug Abuse Modified Alcohol, Smoking and Substance Involvement Screening Test, *CESD-10* Center for Epidemiologic Studies Depression 10-item scale

### Predictors of PrEP nonadherence

The linear-by-linear test of association for AUDIT-based classification was significant, demonstrating that increasing alcohol risk severity was associated with greater occurrence of PrEP nonadherence (i.e., low risk drinkers = 15.6% nonadherent; hazardous drinkers = 25.0% nonadherent; harmful drinkers = 44.4% nonadherent) (χ^2^(1) = 4.79, *p* = .029). Additionally, as shown in Table 3, univariate logistic regression analyses demonstrated that AUDIT-based classification, moderate/high risk cocaine use, and engaging in condomless sex with an HIV-positive partner during the past 3 months were associated with PrEP nonadherence. Associations between depression-related indicators and PrEP nonadherence were not significant. Inclusion of the three significant univariate factors in a multivariate logistic regression model demonstrated that AUDIT-based classification and moderate/high risk cocaine use remained as significant independent predictors, whereby harmful drinkers were over six times more likely to be nonadherent to PrEP than low risk drinkers (AOR = 6.72, 95%CI = 1.49–30.33, *p* = .013), and moderate/high risk cocaine users were over three times more likely to be nonadherent than those without such risk (AOR = 3.11, 95%CI = 1.01–9.59, *p* = .049).

**Table 3:** Associations with PrEP nonadherence in the past 4 days: Univariate and multivariate logistic regression

	PrEP Adherence – Past 4 Days					
	Adherent (*n* = 113)	Nonadherent (*n* = 28)				
Factor	n (%)	n (%)	OR (95% CI)	*p*	AOR (95% CI)	*p*
Age ≥ 35	65 (57.5%)	15 (53.6%)	0.85 (0.37–1.96)	.706		
Race/Ethnicity = white	73 (64.6%)	16 (57.1%)	0.73 (0.32–1.70)	.465		
Education = completed college/university	89 (78.8%)	25 (89.3%)	2.25 (0.63–8.08)	.215		
Annual household income ≥ CAD $100,000	51 (47.2%)	12 (46.2%)	0.96 (0.41–2.26)	.922		
Employed full-time	92 (81.4%)	22 (78.6%)	0.84 (0.30–2.32)	.732		
Taking PrEP for ≥12 months	56 (49.6%)	15 (53.6%)	1.17 (0.51–2.69)	.704		
Currently have a steady partner	43 (38.1%)	12 (42.9%)	1.22 (0.53–2.83)	.641		
Condomless sex with any HIV-negative partners – past 3 months	90 (81.8%)	19 (70.4%)	0.53 (0.20–1.38)	.191		
Condomless sex with any HIV status unknown partners – past 3 months	73 (67.0%)	15 (55.6%)	0.62 (0.26–1.45)	.269		
Condomless sex with any HIV positive partners – past 3 months	46 (41.4%)	16 (59.3%)	2.06 (0.87–4.84)	.099	2.22 (0.89–5.56)	.089
Alcohol Consumption (AUDIT)				.096		.044
Low risk	81 (71.7%)	15 (53.6%)	Ref		Ref	
Hazardous	27 (23.9%)	9 (32.1%)	1.80 (0.71–4.58)	.217	1.54 (0.57–4.18)	.399
Harmful	5 (4.4%)	4 (14.3%)	4.32 (1.04–17.97)	.044	6.72 (1.49–30.33)	.013
Cannabis - moderate/high risk use (NIDA M-ASSIST)	35 (31.0%)	9 (32.1%)	1.06 (0.44–2.57)	.905		
Cocaine - moderate/high risk use (NIDA M-ASSIST)	11 (9.7%)	7 (25.0%)	3.09 (1.07–8.90)	.037	3.11 (1.01–9.59)	.049
Inhalants - moderate/high risk use (NIDA MASSIST)	6 (5.3%)	4 (14.3%)	2.97 (0.78–11.36)	.111		
Sedatives - moderate/high risk use (NIDA MASSIST)	6 (5.3%)	3 (10.7%)	2.14 (0.50–9.15)	.305		
Any substance - moderate/high risk use (NIDA MASSIST)	46 (40.7%)	15 (53.6%)	1.68 (0.73–3.86)	.221		
Currently experiencing depression (CESD-10)	25 (22.5%)	8 (28.6%)	1.38 (0.54–3.50)	.503		
Received treatment for depression – past 3 months	22 (21.6%)	3 (12.0%)	0.50 (0.14–1.81)	.289		

Percentages are based on the number of participants who indicated a specific response divided by the number of participants who responded to the item

ORs could not be calculated for moderate/high risk methamphetamine, hallucinogen, stimulant, street opioid, and prescription opioid use due to empty cells

*AUDIT* Alcohol Use Disorders Identification Test, *NIDA M-ASSIST* National Institute on Drug Abuse Modified Alcohol, Smoking and Substance Involvement Screening Test, *CESD-10* Center for Epidemiologic Studies Depression 10-item scale

### Tests for syndemic effects on PrEP nonadherence

**Fig. 1:**
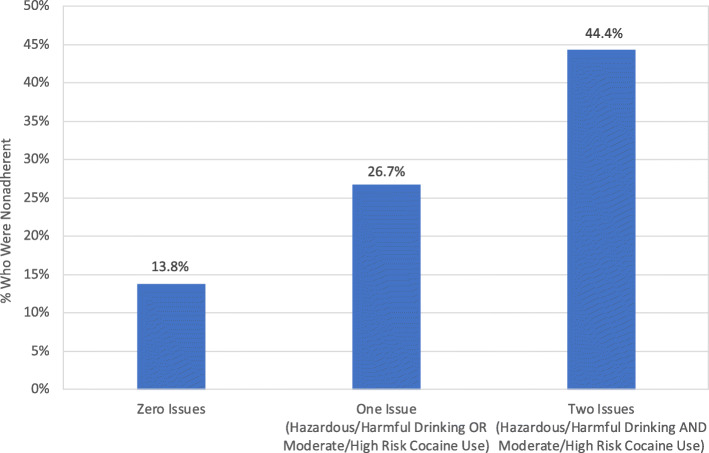
Percent of participants who reported PrEP nonadherence among those with 1) neither hazardous/harmful drinking nor moderate/high risk cocaine use; 2) either hazardous/harmful drinking or moderate/high risk cocaine use; and 3) both hazardous/harmful drinking and moderate/high risk cocaine use

Based on findings from the multivariate logistic regression analysis, additive and interactive effects involving hazardous/harmful alcohol consumption and moderate/high risk cocaine use in relation to PrEP nonadherence were examined. Among the sample of 141 PrEP-prescribed gbMSM, 87 (61.7%) reported neither hazardous/harmful drinking nor moderate/high risk cocaine use; 45 (31.9%) reported one of these issues; and nine (6.4%) reported both issues. As depicted in Fig. 1, the likelihood of PrEP nonadherence increased in conjunction with the number of issues one was experiencing. Accordingly, in support of an additive effect, logistic regression demonstrated that there was an approximately two-fold increase in the likelihood PrEP nonadherence for each additional issue that a participant was experiencing (OR = 2.25, 95%CI = 1.19–4.25, *p* = .013). The interaction between hazardous/harmful drinking and moderate/high risk cocaine use was not significant (OR = 0.77, 95%CI = .090–6.54, *p* = .809), suggesting that these two factors did not work in a synergistic, multiplicative manner in relation to PrEP nonadherence.

## Discussion

One in five participants who had been prescribed daily PrEP for HIV prevention reported missing one or more of their PrEP doses over the past 4 days. Increasingly problematic levels of alcohol consumption among PrEP-prescribed gbMSM were associated with a significantly increased likelihood of PrEP nonadherence, and this association was sustained when accounting for other relevant factors. Moderate/high risk cocaine use was also significantly linked to missing one’s PrEP doses, but indicators related to depression were not. Finally, while problematic alcohol consumption and moderate/high risk cocaine use were shown to have independent and additive associations with PrEP nonadherence, evidence for a synergistic impact of the two risk factors on adherence was not yielded.

Findings pertaining to alcohol use and PrEP nonadherence accord with the broad literature on alcohol and adherence to ART [29, 31], and are consistent with the positive associations demonstrated among some investigations on PrEP adherence and problematic alcohol consumption [32, 34, 35]. In particular, our investigation demonstrated that participants with the most pronounced alcohol problems (i.e., harmful drinkers) were over six times more likely to be nonadherent to PrEP. Consuming alcohol at such problematic levels may substantially hinder one’s motivation and/or ability to take PrEP as prescribed. It may also be the case that perceptions surrounding interactive toxicities between alcohol and PrEP result in conscious decisions to miss one’s doses. Supportive evidence for such a connection has been demonstrated with respect to ART adherence [57], and recent PrEP-focused research suggests that this pattern may similarly exist for PrEP adherence as well [58]. Regardless of the potential mechanism, the demonstrated association between problematic alcohol use and PrEP nonadherence suggests that alcohol may comprise a considerable barrier to PrEP treatment.

Of note is that discrepancies between the current positive alcohol-related findings and the null associations yielded through some previous investigations (e.g., [36, 37]) may relate in part to the manner in which alcohol concerns were operationalized. Specifically, whereas many of these previous studies focused on “heavy drinking” which was typically defined as having a binge drinking episode, the present study employed AUDIT-based drinking criteria, which perhaps is better suited for more reliably identifying those with marked, persistent alcohol problems. Such problems, in turn, may be much more detrimental to PrEP-taking efforts than having an occasional, or even a single, binge drinking occurrence.

With respect to substances, consistent with previous research (e.g., [42]), moderate/high risk cocaine use was significantly associated with PrEP nonadherence. Consuming cocaine at elevated levels may be linked to impaired neurocognitive functioning and pronounced lifestyle disruptions [59]; both of which can diminish the likelihood of following one’s PrEP regimen as prescribed. While this suggests that similar links between the risky use of other stimulants/club drugs (e.g., methamphetamines) and nonadherence should also have been yielded in our investigation [60], it is important to note that infrequent reports of moderate/high risk use of these other substances precluded us from being able to statistically evaluate these associations. Nevertheless, the strong association between cocaine and nonadherence highlights the challenge that substance use poses within the context of PrEP treatment.

Importantly, syndemic-focused analyses demonstrated a significant, progressively increasing likelihood of PrEP nonadherence in correspondence with the increased presence of problematic alcohol and cocaine use. Specifically, although the combined presence of problematic alcohol and cocaine use did not produce a synergistic association (possibly due to statistical power concerns – see below), PrEP nonadherence was significantly most pronounced among those who were experiencing both issues. This additive pattern aligns with the ART adherence-focused literature on syndemics [46, 47], and it builds on this work by providing strong evidence for the dual burden of comorbid alcohol and substance use on PrEP treatment. Recognizing this pattern, and given the prevalence and concurrency of these issues among gbMSM in general [39, 40], it is clear that comorbid alcohol and other substance use require particular attention in PrEP-delivery settings.

Depression-related indicators were not found to be significantly related to PrEP nonadherence. Previous findings linking depression with PrEP adherence have been mixed [45, 61]; leading to the supposition that depression may not play a critical role in one’s PrEP-taking behaviors [45]. Nevertheless, the noteworthy rate of depressive symptomatology reported by our PrEP-prescribed gbMSM suggests that efforts are necessary to identify and address depression among this population.

Taken together, findings from the present investigation have direct implications for PrEP treatment among gbMSM. Our work strongly suggests the need to integrate alcohol- and substance (i.e., cocaine)-focused initiatives into the context of PrEP clinical care. To start, screening for alcohol- and substance-related issues should occur not only during one’s initial PrEP consultation, but also at each PrEP follow-up visit. Such a protocol would ensure that any alcohol and/or substance use issues that persist, develop, or recur over the course of one’s PrEP treatment could be promptly identified. Subsequently, individuals who are recognized as possessing a marked issue regarding their alcohol and/or substance use could then be offered a wide-range of tailored, evidence-based interventions, which would help them achieve their harm-reduction goals, and in turn, could also potentially lead to improvements in PrEP adherence (see [62] for similar effects with respect to ART adherence). Alternatively, PrEP-focused adherence interventions could be offered to PrEP-prescribed gbMSM who possess alcohol- and/or substance-related issues. This targeted approach would be especially appealing in resource constrained settings, as focusing specifically on this particular subgroup that is most at risk for missing PrEP doses would help maximize financial and temporal efficiencies of adherence promotion efforts.

Findings should be viewed in terms of possible limitations. First, our PrEP nonadherence outcome was based on a self-report measure that focused on missing one or more doses over the course of a recent, relatively brief time period (i.e., the past 4 days). In general, self-report PrEP adherence measures have been shown to be subject to a greater degree of underreporting nonadherence compared to objective biomarker tests (e.g., [63–65]). Additionally, given that survey administration was temporally linked to one’s clinic appointment, reports of PrEP adherence may have to some extent been impacted by a white coat compliance effect, whereby adherence to one’s regimen tends to be higher in the days immediately preceding one’s clinic visit [66]. The recency and brevity of our adherence assessment timeframe may therefore have produced adherence reports that may not have fully reflected longer term PrEP adherence patterns among the sample. Of additional relevance regarding our adherence measure was that nonadherence was defined as missing one or more PrEP dose over the past 4 days. This classification therefore included those who missed only one of four doses; reflecting an adherence rate of 75%. Given that some evidence suggests that sufficient protection from HIV acquisition can be obtained at a PrEP adherence rate of 57% (i.e., four out of seven doses per week), our strict definition of nonadherence may possess a relatively lower degree of clinical consequence compared to approaches that employ more lenient adherence metrics.

Second, our assessments of alcohol consumption, substance use, and depression were based on self-report measures. More objective tests for alcohol and other substance use [67–69], along with a clinical diagnosis of depression, would more accurately identify these concerns. Third, data were cross-sectional in nature, and assessment timeframes varied across the validated measures (e.g., 4-day adherence, 3-month substance use). Employing longitudinal approaches and adapting the validated measures to follow consistent response timeframes (e.g., all based on 3 months) would help evaluate the temporal nature of the associations under investigation. Fourth, participants were recruited through convenience sampling at two clinic sites, and the resultant sample reflected a male, highly educated, and high-income population; all of whom had been taking PrEP for at least 3 months. As such, findings from the current investigation may not generalize to the broader PrEP-prescribed population, including women; individuals from less affluent and less educated communities; and those with less established PrEP histories. Finally, the sample was relatively small, which may have resulted in diminished statistical power. This may have limited the ability not only to assess the associations between the moderate/high risk use of specific substances and PrEP nonadherence, but also to test for interactive syndemic effects. Future investigations involving larger samples would potentially be better positioned to evaluate these aspects.

## Conclusions

The present findings underscore the marked role of problematic alcohol consumption and cocaine use in PrEP treatment. Identifying and addressing these issues within PrEP-delivery settings could enhance the effectiveness of PrEP, which in turn could lead to a reduction in HIV incidence among the broader population of gbMSM.

## Data Availability

The datasets generated during and/or analyzed during the current study are not publicly available due to research ethics-related requirements but may be available from the corresponding author on reasonable request.

## Abbreviations

ACTG: AIDS Clinical Trials Group
AOR: Adjusted Odds Ratio
ART: Antiretroviral therapy
AUDIT: Alcohol Use Disorders Identification Test
CESD-10: Center for Epidemiologic Studies Depression 10-item scale
gbMSM: Gay, bisexual and other men-who-have-sex-with-men
HIV: Human Immunodeficiency Virus
NIDA M-ASSIST: National Institute on Drug Abuse Modified Alcohol, Smoking and Substance Involvement Screening Test
OR: Odds Ratio
PrEP: Pre-Exposure Prophylaxis
RCT: Randomized controlled trial
TAF/FTC: Tenofovir alafenamide/emtricitabine
TDF/FTC: Tenofovir disoproxil fumarate/emtricitabine
